# Interval Cancers in a Population-Based Screening Program for Colorectal Cancer in Catalonia, Spain

**DOI:** 10.1155/2015/672410

**Published:** 2015-02-24

**Authors:** M. Garcia, X. Domènech, C. Vidal, E. Torné, N. Milà, G. Binefa, L. Benito, V. Moreno

**Affiliations:** ^1^Cancer Prevention and Control Program, Catalan Institute of Oncology, IDIBELL, L'Hospitalet de Llobregat, 08908 Barcelona, Spain; ^2^Barcelona Health Region, CatSalut, 08023 Barcelona, Spain; ^3^Consortium for Biomedical Research in Epidemiology and Public Health (CIBEResp), 28029 Madrid, Spain; ^4^Department of Fundamental Care and Medical-Surgical Nursing, University of Barcelona, L'Hospitalet de Llobregat, 08907 Barcelona, Spain; ^5^Department of Clinical Sciences, University of Barcelona, L'Hospitalet de Llobregat, 08907 Barcelona, Spain

## Abstract

*Objective*. To analyze interval cancers among participants in a screening program for colorectal cancer (CRC) during four screening rounds. *Methods*. The study population consisted of participants of a fecal occult blood test-based screening program from February 2000 to September 2010, with a 30-month follow-up (*n* = 30,480). We used hospital administration data to identify CRC. An interval cancer was defined as an invasive cancer diagnosed within 30 months of a negative screening result and before the next recommended examination. Gender, age, stage, and site distribution of interval cancers were compared with those in the screen-detected group. *Results*. Within the study period, 97 tumors were screen-detected and 74 tumors were diagnosed after a negative screening. In addition, 17 CRC (18.3%) were found after an inconclusive result and 2 cases were diagnosed within the surveillance interval (2.1%). There was an increase of interval cancers over the four rounds (from 32.4% to 46.0%). When compared with screen-detected cancers, interval cancers were found predominantly in the rectum (OR: 3.66; 95% CI: 1.51–8.88) and at more advanced stages (*P* = 0.025). *Conclusion*. There are large numbers of cancer that are not detected through fecal occult blood test-based screening. The low sensitivity should be emphasized to ensure that individuals with symptoms are not falsely reassured.

## 1. Introduction

Colorectal cancer (CRC) is the third most common cancer and the fourth leading cause of cancer death worldwide. About 1.36 million people are diagnosed annually with CRC, and approximately 694,000 die from CRC annually [1]. Approximately 54% of CRC cases are diagnosed in developed countries, and Europe represents one of the regions with the highest rates in both incidence and mortality. As the sojourn time for CRC is several years and a good prognosis is associated with diagnosis at early stage, screening has been implemented in many countries [2]. The rationale behind cancer screening programs is that early detection of cancer (before symptoms arise) will reduce cause-specific mortality [3]. Compelling and consistent evidence from randomised controlled trials shows that fecal occult blood test and flexible sigmoidoscopy reduce CRC mortality [4, 5]. However, screening also has the potential to harm. The relationship between benefits and risks depends on the quality of screening [6, 7]. The monitoring of interval cancers (IC) is a crucial part of the evaluation of a CRC screening program and a key performance indicator. It provides a mechanism to evaluate the likely impact of the program on CRC mortality in the target population. IC are those that occur following a negative screening episode, in the interval before the next invitation to screening is due [8]. For fecal occult blood testing IC may occur following a negative test, or following a positive test result with a negative further assessment (colonoscopy) [9].

Although IC are inevitable in a screening program, their number should be as small as possible since a high proportion would decrease screening effectiveness. Four plausible reasons (missed polyps or CRC, incompletely resected polyps, rapid progression of new polyps, and failure of biopsy to diagnose a CRC that was present) have been proposed to explain IC [10]. Two studies concluded that 50% to 75% of interval CRC were likely the result of missed or incompletely resected lesions and less than 30% were the result of rapidly progressing lesions [11, 12].

Calculation of the IC rate also allows the calculation of other performance indicators, such as the proportional incidence and program sensitivity. The proportional incidence method compares the incidence rate of IC in successive periods after a negative screen with the expected incidence in the absence of screening. The difference between the two rates gives the number and proportion of CRC whose diagnosis has been advanced by screening [13]. On the other hand, program sensitivity (traditional method) compares screen-detected CRC (SD) with IC. Such a method implies some degree of overestimation of sensitivity, particularly when determined at the first prevalence screening [14]. Proportional incidence also has its limitations, mainly for the difficulty of estimating underlying incidence in absence of screening (i.e., when a cancer registry is lacking or when screening has been ongoing since a long time) [14].

The aim of this study was to analyze IC among participants in the screening program for CRC of L'Hospitalet de Llobregat during four screening rounds. As a secondary objective, program sensitivity was analyzed according to demographic, screening, and tumor characteristics.

## 2. Material and Methods

### 2.1. Screening Procedure

In 2000, a biennial screening program was launched in L'Hospitalet of Llobregat, an industrial city in the metropolitan area of Barcelona (Catalonia, Spain). The target population includes all men and women aged 50–69 years who lived in the city (average of 65,000). Demographic data on this population is gathered from the Primary Healthcare Information System. L'Hospitalet of Llobregat is divided into 12 Basic Health Areas and screening invitations are sent to eligible population assigned to each one of the Basic Health Areas. Subjects who do not meet the inclusion criteria for CRC screening are definitely or temporarily excluded according to the following criteria: personal history of CRC or adenomas, hereditary and familial CRC, inflammatory bowel disease, colonoscopy in the previous 5 years, fecal occult blood test in less than 2 years, terminal disease, and severe disabling condition. Subjects moving out of the screening area or whose invitation letter is returned because of an invalid mailing address are also excluded.

CRC screening criteria are assessed by means of a questionnaire. When the questionnaire reports two or more relatives with cancer, individuals are phoned through the program to check the information and evaluate whether he/she is eligible for CRC screening with fecal occult blood testing or met the criteria for hereditary colorectal cancer. If a high risk family history was confirmed, the individual is excluded from the program and is referred to a genetic counseling unit for a more detailed assessment. The program allows the screening of individuals with a family history of CRC or other noncolonic neoplasms as long as they do not meet the criteria for hereditary cancer.

Since 2000, two screening test strategies have been used. From the first to third rounds, a guaiac FOBT (gFOBT) was used as the screening test (hema-screenTM, Immunostics Inc., Ocean, NJ, USA). In the fourth round, the gFOBT was offered to 50,227 individuals (eligible population assigned to 10 Basic Health Areas) and a quantitative immunochemical test (OC-Auto Sampling Bottle 3, Eiken Chemical Co., Japan) was offered to 12,707 (eligible population assigned to two Basic Health Areas). The immunochemical test (FIT) was initially introduced to the screening program to evaluate its feasibility and acceptability. Briefly, participation was higher among individuals who used the FIT (OR: 1.35; 95% CI: 1.27–1.42). Detection rates for adenomas and cancer were also higher for the FIT, highlighting the detection rate for high-risk adenomas (26.7‰ versus 3.0‰). The positive predictive value for high-risk adenomas was quite similar (45.0% and 46.9% in the FIT and gFOBT, resp.) [15]. As a result, FIT remained the only strategy for further screening rounds (fifth round and onwards).

Participants with gFOBT collected six fecal samples (two samples from three separate bowel movements). The gFOBT uses a chemical indicator that shows a color change in the presence of blood. The possible results of the gFOBT were (a) weakly positive: one to four positive samples. Those participants with a weakly positive result were asked to perform a second gFOBT and, if any sample was positive, were offered colonoscopy without further testing. In contrast, if all six samples were negative, a third gFOBT was requested; (b) spoilt kit/technical failure: laboratory was unable to analyze the kit. The most common reason for a rejected kit was that the information provided with the kit was not complete. Those participants who refused to repeat the test after a weak positive or a spoilt kit/technical failure were coded with an indeterminate gFOBT result; (c) negative: zero out of six positive samples; (d) strongly positive: five or six positive samples.

On the other hand, participants with FIT collected one sample (approximately 10 mg of feces) which was added to 2 mL buffer. A 100 ng Hb/mL cut-off (20 mg Hb/g feces) was used as threshold for test positivity. Tests were assayed generally on the day of receipt in the laboratory on one of two automated clinical analyzers (OC-Sensor Micro or OC-Sensor Diana). Samples were at 2°–8°C if not analyzed on the day of receipt and then allowed to warm to room temperature for the assay. Each sample was analyzed once. The upper limit of the analytical working range for the fecal Hb concentration measurements was 1,000 ng Hb/mL buffer (200 mg Hb/g feces); samples with concentrations greater than this were not diluted and not reassayed. When laboratory was unable to analyze a test (spoilt kit or technical failure), the participant was asked to repeat the FIT. If she/he refused, then the final test result was coded as an indeterminate.

All participants with a positive test result were advised to have colonoscopy. Subjects with no colorectal lesions detected in the colonoscopy are invited for screening again after 10 years (if they are still within the target age group).

### 2.2. Study Population

The study population consisted of participants of a CRC screening program from February 2000 to September 2010, with a minimum of 30-month follow-up (*n* = 30,480). The period of study included four rounds of the CRC screening program.

### 2.3. Data Collection and Variables

The CRC screening program information system included data on individual identification, age, gender, participation, appointment dates, screening test (guaiac or immunochemical), final screening test results (positive, negative, or indeterminate), and colonoscopy results (negative, precursor lesions, and CRC).

We used hospital administration data (minimum data set (MDS)) to identify CRC. A SD was defined as an invasive CRC diagnosed at colonoscopy triggered by a final positive screening test result. On the other hand, IC was defined as an invasive CRC diagnosed following a negative screening episode and prior to the next scheduled screening examination. The next scheduled screening examination was defined to be 30 months after the previous screen. Screening interval was 24 months; however, it should be considered adequate up to 30 months (acceptable delay because of organizational and management issues). Our screening program was launched as a pilot program and was considered an established program by the third round. The median time between invitations was 33 months (higher in the earlier rounds and descending in the subsequent rounds).

Electronic medical records of the individuals identified as being diagnosed for CRC were revised to gather tumor characteristics, for instance, the anatomic pathology result of the cancerous lesion and the extension study. CRC were staged according to the tumor-nodal-metastasis (TNM) staging system and classified as early (TNM I/II) or late (TNM III/IV) stage. Tumor site was grouped in three categories: proximal defined as the region of the colon up to and including the splenic flexure, distal including the descending and sigmoid colon, and rectum.

Patients were classified into 4 groups according to participation in the screening program: (1) individuals with CRC diagnosed by screening; (2) individuals with a negative screening result and CRC diagnosed prior to further screening round; and (3) individuals with an incomplete screening process: (a) indeterminate test, (b) screenees with a positive test who did not attend the colonoscopy and were diagnosed during the interval as they became symptomatic; (4) individuals clinically diagnosed with CRC after 30 months of their last screening (mainly nonattenders in further screening rounds).

The program sensitivity was expressed as the number of SD divided by both SD and IC following a normal screen or assessment (traditional method).

The study protocol was approved by the Clinical Research Ethics Committee of the Bellvitge University Hospital and all involved parties followed the ethical requirements set forth in the Spanish Organic Law on Protection of Personal Data (15/1999 of December 13).

### 2.4. Statistical Analysis

A descriptive analysis of all screenees diagnosed with CRC was carried out. Program sensitivity was calculated according to demographic, screening, and tumor characteristics.

Factors associated with IC were analyzed using multivariate logistic regression models. The variables included in the multivariate analysis were gender, age, number of screens, tumor site, and CRC stage. The results were expressed as odds ratios (OR) with 95% confidence intervals (95% CI). Differences were considered statistically significant when *P* < 0.05. All analyses were performed using R statistical software (R Foundation for Statistical Computing, Vienna, Austria).

## 3. Results

**Figure 1:**
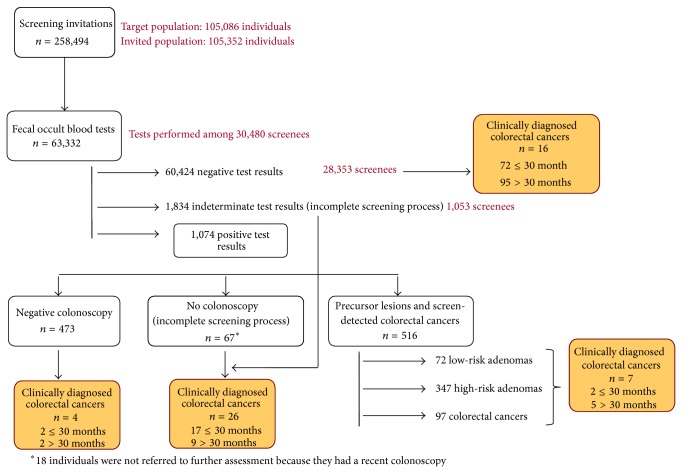
Flowchart of the screening process from 2000 to 2010.

Within the study period, 301 CRC were diagnosed in the screened population (Figure 1). From those, 97 tumors were detected during the screening process and 93 tumors were diagnosed within 30 months after the screening examination. IC were diagnosed after a negative result in the screening process (*n* = 74). In addition, 17 clinically diagnosed CRC (18.3%) were found after an inconclusive result and 2 cases were diagnosed within the surveillance interval (2.1%).

On the other hand, 111 CRC were found among symptomatic individuals who did not attend screening in further rounds; they were diagnosed with CRC after 30 months of their last screening (range from 31 months to 12.2 years). From those, 97 completed the screening process and had a negative result.

Around 85% of the screenees used the gFOBT as the screening strategy and the remaining 15% used the FIT (Table 1). Regarding the clinically diagnosed tumors, eight CRC were diagnosed in individuals who used the FIT and half of the CRC were detected within 30 months after their participation in the screening (Table 2).

The overall program sensitivity was 56.7% and was higher among men and young individuals (Table 3). Regarding the test used, sensitivity was 52.0% with gFOBT and 87.0% with FIT.

Table 4 reports the results of the multivariate logistic regression model. When compared with SD, IC were predominantly in the rectum (OR: 3.66; 95% CI: 1.51–8.88). When stratified by gender, no differences in cancer site were found. This held for both SD and IC (Table 5).

Finally, differences in cancer staging were observed. Individuals with a CRC diagnosed within 30 months after their last screening had more advanced stages (*P* = 0.025).

## 4. Discussion

The overall program sensitivity was relatively low. A high false negative rate should be emphasized to clinicians and patients to ensure that those with symptoms are not falsely reassured and slip through the diagnostic net [16].

Program sensitivity estimated in randomized clinical trials ranged from 38% to 54% [17]. Some population-based screening programs for CRC have also calculated program sensitivity. Those programs using gFOBT reported an overall sensitivity from 42.3% to 62.4% [18–22].

Otherwise, sensitivity was higher in those programs using FIT (from 71.3% to 85.6%) [23, 24]. It is worth mentioning that program sensitivity decreases as more screening rounds are included in the study. Although in our study FIT sensitivity was considerably higher compared to gFOBT sensitivity (87.0% versus 52.0%, resp.), we have to be cautious due to the small sample size of screenees using FIT.

Around 18% of CRC were identified in individuals who did not complete the screening process because of an inconclusive FOBT or refusal of further assessment (colonoscopy). Strategies aimed at eliminating or reduce inconclusive test results should be implemented. In this way, both screening quality and program sensitivity would improve.

Efforts in communicating the need for being screened regularly should be made. One-third of CRC identified among individuals who had ever participated in the CRC screening program were diagnosed after 30 months of their last screening. Once in a lifetime screening is not enough, especially if the screening modality is FOBT. On the contrary, successive screening is needed to detect and prevent CRC. The sojourn time (the period when a test can detect asymptomatic disease) is being estimated in 2.2 to 4.9 years for gFOBT [25, 26].

However, some of the CRC clinically diagnosed among individuals after 30 months of their last screening could be averted if they would have been invited within an adequate screening interval. Time between invitations from the early rounds (first and second screening round) was on average 36 months due to some management and organizational concerns. By the third round, time between invitations was acceptable.

Regarding factors associated with IC, differences in sensitivity according to tumor site and cancer stage have been found. We did not find differences in the IC according to gender. However, some studies have shown higher IC rates among women [21, 22, 27, 28].

Most of the studies have shown that IC were more frequently located in the proximal colon [18, 22, 27]. Tazi et al. found IC were more likely to arise in the rectum compared with SD [19].

Finally, our findings regarding IC being diagnosed at more advanced stages are consistent with the literature [19, 20, 22, 27, 28].

Among the strengths of this study it is worth mentioning that it is the first study to report IC in a population-based screening program for CRC in Spain. In addition, the length of the study period allows estimating program sensitivity in both prevalent and incident rounds. Most of the studies aimed at analyzing IC have included only two rounds.

Some of the limitations of this study are related with case ascertainment and completeness of cancer data. Interval cancers should be ideally identified through a cancer registry. Checking hospital discharge records may be very useful in areas uncovered by a cancer registry as in our case. However, such a method tends to ignore cases treated outside the National Health System and/or having no hospitalization and those who migrate to a different region. In our study, 85% of CRC diagnosed in private hospitals could be identified. However, there was some missing information regarding tumor characteristics such as the cancer site or stage.

We could not perform a multivariate logistic regression analysis stratified by the test used (gFOBT or FIT) because of the small number of cases. However, as the FIT remained the only one screening strategy by fifth round and onwards, we will be able to evaluate FIT sensitivity and identify whether there are differences according to demographic, screening, and tumor characteristics.

As far as we know, only one study in Spain validated the ability of hospital administration data set to detect incident cases of CRC using a cancer registry as the gold standard and measured agreement between the MDS and registration of CRC [29]. The study population consisted of incident cases of CRC in 2000 obtained from the cancer registry and cases in the MDS of a public hospital for the same year. Around 2% of CRC identified through the cancer registry did not require hospitalization. The MDS detected 85% of the cases and the main reason to not identify cases was differences regarding the incidence date (12 out 13 cases that did not match were found in 2001). We revised CRC from 2000 to 2013, so this potential error should be minimized.

Another limitation is that we calculated the program sensitivity using the traditional method and without taking into account the CRC sojourn time. We could not calculate proportional incidence because we do not have a cancer registry that covers our screening area. However, the program sensitivity may be more easily interpretable than the proportional incidence and is not dependent on the underlying incidence of disease in the screened population.

### 4.1. Future Research

Further research is needed to discriminate IC due to a false negative screening result from interval cancers* de novo*, with a rapid tumor growth. Recent biological data have begun to suggest that a proportion of IC exhibits altered biological features that may contribute to their rapid development relative to those that are detected on screening [30]. Thus, it remains necessary to further establish and expand our understanding of molecular pathways involved in IC to modify and/or develop appropriate screening programs and specific treatment options to combat this unique form of CRC [31, 32].

Association of molecular features (i.e., BRAF and KRAS mutations, CpG island methylator phenotype, and sporadic microsatellite instability) with dietary and lifestyle factors also needs to be explored [33, 34].

### 4.2. Methodological Issues for Benchmarking and Comparison of IC

There is some variation regarding how IC are actually defined, identified, and reported in different programs. Differences in definition, quantification, or completeness of identification of IC between programs distort the underlying differences in IC frequency and obscure interpretation of this measure [35].

Operational definition and quantification method for interval cancer are needed to eliminate or control some sources of artefactual variation across programs. The accuracy of identification of interval cancer may potentially be the largest source of error and discrepancy between programs [35].

There are a few expert working groups that are making efforts to that end (NHS Bowel Cancer Screening Program, World Endoscopy Organization, Spanish Network of Cancer Screening Programs) [36, 37]. Their aim is to define IC to facilitate benchmarking and comparison of IC rates across programs and regions. Incomplete follow-up in the screened population and imprecise linkage between data sources are the main limiting factors in the identification of IC.

## 5. Conclusion

There are large numbers of cancer that are not detected through fecal occult blood test-based screening. Screening programs using gFOBT should take into account that initiatives aimed at decreasing inconclusive results could improve screening quality and program sensitivity. Nevertheless, many European countries that have introduced population-based screening programs based on gFOBT have already switched to immunoassay as their screening test of choice. As a consequence, there are fewer individuals using FIT with an incomplete screening episode and the overall sensitivity is higher compared with gFOBT screening programs.

On the other hand, high CRC rates among nonattenders in further screening rounds highlight the need to better inform our target population that successive screening is required to avert or early detect CRC.

Variation in calculating the interval cancer rate makes it vital that a clearly defined protocol is established for the definition, identification, and reporting of interval cancers. International comparisons and benchmarking of IC could lead to better understanding of the relationship between programs performance and screening practices.

## Figures and Tables

**Table 1:** Last screening result according to the test used (gFOBT or FIT)^‡^ from 2000 to 2010.

	gFOBT		FIT	
	*n*	%	*n*	%
Negative test result	24,072	93.1	4,281	92.7
Indeterminate test result	1,003	3.9	50	1.1
Positive test result	787	3.0	287	6.0
Positive test with no further assessment^*^	58	0.2	9	0.2
Negative colonoscopy result	368	1.4	105	2.3
Low-risk adenoma	48	0.2	24	0.5
High-risk adenoma	225	0.9	122	2.6
CRC	77	0.3	20	0.4
Overall	25,862		4,618	

^‡^gFOBT was the only screening test used in 1st–3rd round. For the fourth round the gFOBT was offered to individuals in 10 Basic Health Areas and the FIT was offered to individuals in two Basic Health Areas; ^*^11 individuals using gFOBT and 7 individuals using FIT were not referred to further assessment because they had a recent colonoscopy.

**Table 2:** Clinically diagnosed CRC among screening participants according to the test used (gFOBT or FIT) and their last screening result.

	CRC diagnosed within ≤30 months			CRC diagnosed >30 months		
	gFOBT	FIT	Overall	gFOBT	FIT	Overall
Negative screening result^*^	71	3	74	95	2	97
Incomplete screening process	16	1	17	9		9
CRC after removal of an adenoma	2		2	3	2	5
Overall	89	4	93	107	4	111

^*^It refers to a negative test or a positive test with a negative colonoscopy result.

**Table 3:** Program sensitivity according to demographic, screening, and tumor characteristics.

	Screen-detected CRC	Interval CRC ≤30 months^‡^	CRC diagnosed >30 months^‡^	Program sensitivity^*^
	*n* (%)	*n* (%)	*n* (%)	%
Gender				
Men	65 (67.0)	47 (63.5)	62 (63.9)	58.0
Women	32 (33.0)	27 (36.5)	35 (36.1)	54.0
Age at diagnosis				
50–59	29 (33.3)	13 (17.6)	5 (5.2)	69.0
60–69	45 (51.7)	54 (73.0)	32 (33.0)	45.5
≥70	23 (14.9)	7 (9.5)	60 (61.9)	76.7
Screening				
First screening	53 (54.6)	26 (35.1)	58 (59.8)	67.1
Successive screenings	44 (45.4)	48 (64.9)	39 (40.2)	47.8
Last screening round				
1	23 (23.7)	11 (14.9)	38 (39.2)	67.6
2	13 (13.4)	14 (18.9)	34 (35.1)	48.1
3	27 (27.8)	20 (27.0)	14 (14.4)	57.4
4	34 (35.1)	29 (39.2)^†^	11 (11.3)^#^	54.0
Number of screens				
1	53 (54.6)	26 (35.1)	58 (59.8)	67.1
2	17 (17.5)	27 (36.5)	30 (30.9)	38.6
3	19 (19.6)	16 (21.6)	7 (7.2)	54.3
4	8 (8.2)	5 (6.8)	2 (2.1)	61.5
Tumor site^**^				
Proximal	23 (23.7)	15 (25.0)	28 (40.0)	60.5
Distal	56 (57.7)	20 (33.3)	24 (34.3)	73.7
Rectum	18 (18.6)	25 (41.7)	18 (25.7)	41.9
Tumor stage^**^				
I	39 (40.2)	9 (14.8)	14 (17.3)	81.3
II	18 (18.6)	17 (27.9)	27 (33.3)	51.4
III	30 (30.9)	28 (45.9)	27 (33.3)	51.7
IV	10 (10.3)	7 (11.5)	13 (16.0)	58.8

^‡^Only CRC detected in individuals with a negative result in their last screening were considered; ^*^sensitivity expressed as the proportion of CRC diagnosed during the screening divided by all CRC diagnosed among screening participants up to 30 months; ^**^variables with missing values; ^†^26 CRC diagnosed after a negative gFOBT and 3 CRC diagnosed after a negative FIT; ^#^9 CRC diagnosed after a negative gFOBT and 2 CRC diagnosed after a negative FIT.

**Table 4:** Factors associated with clinically diagnosed CRC among individuals who ever participated in the screening program for CRC (≤30 months versus >30 months since their last screening).

	Interval CRC ≤30 months		Clinically diagnosed CRC >30 months	
	OR_a_	95% CI	OR_a_	95% CI
Gender				
Men	1		1	
Women	1.02	(0.44–2.33)	1.94	(0.69–5.43)
Age at diagnosis^*^	1.03	(0.96–1.11)	1.38	(1.24–1.53)
Number of screens	1.24	(0.85–1.81)	0.55	(0.32–0.94)
Tumor site				
Distal	1		1	
Proximal	1.32	(0.51–3.39)	3.73	(1.21–11.51)
Rectum	3.66	(1.51–8.88)	3.48	(1.02–11.87)
Tumor stage				
I	1		1	
II	2.63	(0.86–8.04)	2.21	(0.61–8.07)
III	3.45	(1.39–8.30)	1.66	(0.51–5.43)
IV	2.80	(0.73–10.76)	4.86	(1.02–23.24)

OR_a_ adjusted odds ratios derived from multivariate logistic regression models. Analysis was restricted to those individuals with a negative result in their last screening that have been diagnosed with CRC later on and those who were diagnosed during the screening process. ^*^Age at diagnosis was considered as a continuous variable; the age range was 50–73 years for individuals with a CRC diagnosed within 30 months after their screening and 55–80 years for individuals who ever participated in the screening program and were diagnosed with CRC.

**Table 5:** Distribution of screen-detected and clinically detected CRC according to tumor site and gender.

Tumor site	Screen-detected CRC		Interval CRC (≤30 months)		Clinically diagnosed CRC >30 months	
	Men	Women	Men	Women	Men	Women
	*n* (%)	*n* (%)	*n* (%)	*n* (%)	*n* (%)	*n* (%)
Proximal	13 (20.0)	10 (31.3)	8 (19.5)	7 (36.8)	16 (37.2)	12 (44.4)
Distal	37 (56.9)	19 (59.4)	14 (34.1)	6 (31.6)	14 (32.6)	10 (37.0)
Rectum	15 (23.1)	3 (9.4)	19 (46.3)	6 (31.6)	13 (30.2)	5 (18.5)

Men versus women in screen-detected CRC: *χ* ^2^ = 3.34, *P* = 0.19; men versus women in interval CRC (≤30 months): *χ* ^2^ = 2.26, *P* = 0.32; men versus women in clinically-diagnosed CRC >30 months: *χ* ^2^ = 1.20; *P* = 0.55.

## References

[1] Ferlay J., Soerjomataram I., Ervik M. (2013). *GLOBOCAN 2012 v1.0, Cancer Incidence and Mortality Worldwide: IARC Cancer Base no. 11*.

[2] Holme Ø., Bretthauer M., Fretheim A., Odgaard-Jensen J., Hoff G. (2013). Flexible sigmoidoscopy versus faecal occult blood testing for colorectal cancer screening in asymptomatic individuals. *The Cochrane Database of Systematic Reviews*.

[3] Van Dam L., Bretthauer M. (2014). Ethical issues in colorectal cancer screening. *Best Practice and Research: Clinical Gastroenterology*.

[4] Hewitson P., Glasziou P., Watson E., Towler B., Irwig L. (2008). Cochrane systematic review of colorectal cancer screening using the fecal occult blood test (Hemoccult): an update. *The American Journal of Gastroenterology*.

[5] Elmunzer B. J., Hayward R. A., Schoenfeld P. S., Saini S. D., Deshpande A., Waljee A. K. (2012). Effect of flexible sigmoidoscopy-based screening on incidence and mortality of colorectal cancer; a systematic review and meta-analysis of randomized controlled trials. *PLoS Medicine*.

[6] Gray J. A. M., Patnick J., Blanks R. (2008). Maximising benefit and minimising harm of screening. *British Medical Journal*.

[7] Prevost T. C., Launoy G., Duffy S. W., Chen H. H. (1998). Estimating sensitivity and sojourn time in screening for colorectal cancer: a comparison of statistical approaches. *The American Journal of Epidemiology*.

[8] Robinson M. H. E., Hardcastle J. D., Moss S. M. (1999). The risks of screening: data from the Nottingham randomised controlled trial of faecal occult blood screening for colorectal cancer. *Gut*.

[9] Halloran S., Launoy G., Zappa M., Segnan N., Patnick J., von Karsa L. (2010). Evidence for the effectiveness of colorectal cancer screening. *European Guidelines for Quality Assurance in Colorectal Cancer Screening and Diagnosis*.

[10] Patel S. G., Ahnen D. J. (2014). Prevention of interval colorectal cancers: what every clinician needs to know. *Clinical Gastroenterology and Hepatology*.

[11] Pabby A., Schoen R. E., Weissfeld J. L. (2005). Analysis of colorectal cancer occurrence during surveillance colonoscopy in the dietary Polyp Prevention Trial. *Gastrointestinal Endoscopy*.

[12] Robertson D., Lieberman D., Winawer S. J. (2008). 795 interval cancer after total colonoscopy: results from a pooled analysis of eight studies. *Gastroenterology*.

[13] Moss S. M., Hardcastle J. D., Coleman D. A., Robinson M. H. E., Rodrigues V. C. (1999). Interval cancers in a randomized controlled trial of screening for colorectal cancer using a faecal occult blood test. *International Journal of Epidemiology*.

[14] Ciatto S., Naldoni C., Ponti A. (2008). Interval carcinomas as indicators of screenig programme performance. Evaluation modalities and standards. *Epidemiologia & Prevenzione*.

[15] Martínez M. G., Rodríguez G. B., Díaz N. M. (2011). Evaluating colorectal cancer screening strategies (immunological test vs biochemical test) in Catalonia, Spain 2008–2010. *Revista Española de Salud Pública*.

[16] Hallifax R., Lacey M., Bevis P., Borley N. R., Brooklyn T., Wheeler J. M. D. (2012). Slipping through the bowel cancer screening programme. *Colorectal Disease*.

[17] Medical Advisory Secretariat (2009). Fecal occult blood test for colorectal cancer screening: an evidence-based analysis. Toronto, ON, Canada: Ontario Ministry of Health and Long-Term Care. *Ontario Health Technology Assessment Series*.

[18] Giai J., Exbrayat C., Boussat B. (2014). Sensitivity of a colorectal cancer screening program based on a guaiac test: a population-based study. *Clinics and Research in Hepatology and Gastroenterology*.

[19] Tazi M. A., Faivre J., Lejeune C., Bolard P., Phelip J. M., Benhamiche A. M. (1999). Interval cancers in a community-based programme of colorectal cancer screening with faecal occult blood test. *European Journal of Cancer Prevention*.

[20] Gill M. D., Bramble M. G., Rees C. J., Lee T. J. W., Bradburn D. M., Mills S. J. (2012). Comparison of screen-detected and interval colorectal cancers in bowel cancer screening programme. *British Journal of Cancer*.

[21] Moss S. M., Campbell C., Melia J. (2012). Performance measures in three rounds of the English bowel cancer screening pilot. *Gut*.

[22] Steele R. J. C., McClements P., Watling C. (2012). Interval cancers in a FOBT-based colorectal cancer population screening programme: implications for stage, gender and tumour site. *Gut*.

[23] Zorzi M., Fedato C., Grazzini G. (2011). High sensitivity of five colorectal screening programmes with faecal immunochemical test in the Veneto Region, Italy. *Gut*.

[24] Zappa M., Castiglione G., Paci E. (2001). Measuring interval cancers in population-based screening using different assays of fecal occult blood testing: the district of florence experience. *International Journal of Cancer*.

[25] Launoy G., Smith T. C., Duffy S. W., Bouvier V. (1997). Colorectal cancer mass-screening: estimation of faecaloccult blood test sensitivity,taking into account cancer mean sojourn time. *International Journal of Cancer*.

[26] Zheng W., Rutter C. M. (2012). Estimated mean sojourn time associated with hemoccult sensa for detection of proximal and distal colorectal cancer. *Cancer Epidemiology Biomarkers and Prevention*.

[27] Morris E. J. A., Whitehouse L. E., Farrell T. (2012). A retrospective observational study examining the characteristics and outcomes of tumours diagnosed within and without of the English NHS bowel cancer screening programme. *British Journal of Cancer*.

[28] Paimela H., Malila N., Palva T., Hakulinen T., Vertio H., Järvinen H. (2010). Early detection of colorectal cancer with faecal occult blood test screening. *British Journal of Surgery*.

[29] Cid M. M., Niñirola I. V., López M. D. C., Martínez J. T., Sánchez E. P., Sánchez C. N. (2006). Validation of colorectal cancer diagnostic codes in a hospital administration data set. *Gaceta Sanitaria*.

[30] Richter J. M., Pino M. S., Austin T. R. (2014). Genetic mechanisms in interval colon cancers. *Digestive Diseases and Sciences*.

[31] Cisyk A. L., Singh H., McManus K. J. (2014). Establishing a biological profile for interval colorectal cancer. *Digestive Diseases and Sciences*.

[32] Arain M. A., Sawhney M., Sheikh S. (2010). CIMP status of interval colon cancers: another piece to the puzzle. *The American Journal of Gastroenterology*.

[33] Shaukat A., Arain M., Thaygarajan B., Bond J. H., Sawhney M. (2010). Is BRAF mutation associated with interval colorectal cancers?. *Digestive Diseases and Sciences*.

[34] Shaukat A., Arain M., Anway R., Manaktala S., Pohlman L., Thyagarajan B. (2012). Is KRAS mutation associated with interval colorectal cancers?. *Digestive Diseases and Sciences*.

[35] Bulliard J.-L., Sasieni P., Klabunde C., de Landtsheer J.-P., Yankaskas B. C., Fracheboud J. (2006). Methodological issues in international comparison of interval breast cancers. *International Journal of Cancer*.

[36] Sandelau S. Report of the Expert Working Group ‘Right-sided lesions & Interval CRCs’. http://worldendo.org/assets/downloads/pdf/resources/ccsc/2014/03_Sanduleanu_Right_sided_lesions_and_Interval_Cancers.pdf.

[37] Almazán R. Protocol proposal for the assessment of interval cancers in colorectal cancer screening programs. http://www.programascancerdemama.org/images/archivos/C_8_Protocolo_CI_CCR.pdf.

